# Frequency Effects on Spelling Error Recognition: An ERP Study

**DOI:** 10.3389/fpsyg.2022.834852

**Published:** 2022-04-14

**Authors:** Ekaterina V. Larionova, Olga V. Martynova

**Affiliations:** ^1^Institute of Higher Nervous Activity and Neurophysiology, Russian Academy of Sciences, Moscow, Russia; ^2^Centre for Cognition and Decision Making, National Research University Higher School of Economics, Moscow, Russia

**Keywords:** reading, error recognition, visual word recognition, event related potential, word frequency

## Abstract

Spelling errors are ubiquitous in all writing systems. Most studies exploring spelling errors focused on the phonological plausibility of errors. However, unlike typical pseudohomophones, spelling errors occur in naturally produced written language. We investigated the time course of recognition of the most frequent orthographic errors in Russian (error in an unstressed vowel in the root) and the effect of word frequency on this process. During event-related potentials (ERP) recording, 26 native Russian speakers silently read high-frequency correctly spelled words, low-frequency correctly spelled words, high-frequency words with errors, and low-frequency words with errors. The amplitude of P200 was more positive for correctly spelled words than for misspelled words and did not depend on the frequency of the words. In addition, in the 350–500-ms time window, we found a more negative response for misspelled words than for correctly spelled words in parietal–temporal-occipital regions regardless of word frequency. Considering our results in the context of a dual-route model, we concluded that recognizing misspelled high-frequency and low-frequency words involves common orthographic and phonological processes associated with P200 and N400 components such as whole word orthography processing and activation of phonological representations correspondingly. However, at the 500–700 ms stage (associated with lexical-semantic access in our study), error recognition depends on the word frequency. One possible explanation for these differences could be that at the 500–700 ms stage recognition of high-frequency misspelled and correctly spelled words shifts from phonological to orthographic processes, while low-frequency misspelled words are accompanied by more prolonged phonological activation. We believe these processes may be associated with different ERP components P300 and N400, reflecting a temporal overlap between categorization processes based on orthographic properties for high-frequency words and phonological processes for low-frequency words. Therefore, our results complement existing reading models and demonstrate that the neuronal underpinnings of spelling error recognition during reading may depend on word frequency.

## Introduction

Reading speed and efficiency are achieved through automatic visual word recognition. Reading includes the visual encoding of letters, transforming letters into graphemes and orthographic patterns, lexical and phonological analysis, and understanding the meaning of written words (Grainger and Jacobs, 1996; Bentin et al., 1999). There are two opposing theories: the direct access hypothesis, which assumes that the meaning of a word is accessed directly from orthography (Seidenberg, 1985), and the phonological mediation hypothesis, which assumes that the meaning of a word is accessed from phonology (van Orden, 1987; Frost, 1998). These two hypotheses have been combined into two interacting paths in dual-route theory, according to which both phonological mediation (non-lexical route) and direct orthographic access (lexical route) are possible paths to the meaning of a word: e.g., the meanings of more frequent words are activated by an orthographic strategy, while the meanings of less frequent words are activated by phonological decoding strategies (Coltheart et al., 2001; Harm and Seidenberg, 2004). ERPs are particularly useful in studying the involved ways of visual word recognition allowing us to determine different stages of word processing, and are especially valuable in silent reading paradigms when we cannot measure behavioral indicators. In the present study, we used ERPs to investigate the time course of recognition of spelling errors in Russian and examine if and when word frequency modulates the spelling processing during reading. The objective of this research is to determine whether the process of recognizing errors in words of different frequency is limited to the stage of orthographic processing or involves phonological and semantic processes; we used the effect of frequency as a marker of lexical access.

Spelling errors are typically thought of as a footprint of a word’s weak orthographic representation in the mind, and the cause of their occurrence is a lack of stability in a word’s spelling, sound, or semantics (Perfetti, 1985; Perfetti and Hart, 2001). Most studies on spelling errors focused on the errors’ phonological plausibility (Landerl and Wimmer, 2000; Caravolas and Volín, 2001; Quémart and Casalis, 2017). Some misspelled words are indeed pseudohomophones. Pseudohomophones differ from words in their orthography but have the same phonology, for example, pseudohomophone SPAIS and word SPACE in English. The pseudohomophone effect is a well-studied marker of phonological activation and comprises a higher error rate and response time for pseudohomophones compared to base words; in addition, pseudohomophones are more often falsely classified as words, presumably due to correct phonological representation (Rubenstein et al., 1971; van Orden, 1987; Goswami et al., 2001; Pexman et al., 2001; Briesemeister et al., 2009). Rahmanian and Kuperman (2019) noted that spelling errors are fundamentally different from typical pseudohomophones used in studies: they occur in naturally produced written language. As the use of basic spelling errors as pseudohomophone stimuli is relatively rare and not so many works studied real spelling errors, we have to rely on previous ERP research on pseudohomophones, given the similarity to the type of misspelled words our research focuses on.

Previous studies have shown the effect of frequency on the recognition of pseudohomophones (Jared and Seidenberg, 1991; Braun et al., 2009, 2015, more details about ERP data are discussed below). Basically, word frequency is one of the strongest predictors of word processing efficiency (Monsell et al., 1989; Brysbaert et al., 2018). High-frequency words are familiar to more people and are processed faster than low-frequency words (Monsell et al., 1989). The frequency effect has been identified in many languages: English (Rayner, 1998), Portuguese (Faísca et al., 2019), French (Segui et al., 1982), Chinese (Yan et al., 2006), and Russian (Laurinavichyute et al., 2019). According to the multiple read-out model (MROM; Jacobs et al., 1998) and the dual-route cascaded model (DRC; Coltheart et al., 2001), high-frequency words have higher summed global lexical activity than low-frequency words. Therefore, one can expect that pseudohomophones, pseudowords, and probably also misspelled words created from a high-frequency word should elicit higher activation resulting in more errors and slower reaction time. However, experimental data for mean reaction time did not support this assumption (Ziegler et al., 2001; Reynolds and Besner, 2005). The seeming contradiction can be explained by assessing the reaction time distributions: high-frequency pseudowords produced slower latencies in the leading edge of the reaction time distributions, which indicates the influence of the early activation process on fast responses and is consistent with MROM and DRC predictions (Perea et al., 2005). Close to the activation-based models is the hypothesis that the uncertainty created by competition between different spellings will be greater for high-frequency words since high-frequency words afford more opportunities to encounter all their spelling variants (Rahmanian and Kuperman, 2019). Also, important to note in particular, for the Russian language, that high-frequency words are more prone to phonetic changes, and some reduced realizations have become so typical that they occur even in written discourse (Bondarko et al., 1988; Raeva and Riekhakaynen, 2016). Such lack of stability in a word’s sound may be the cause of the occurrence of spelling errors (Perfetti, 1985; Perfetti and Hart, 2001). Therefore, error recognition in words of different frequencies may differ, and thus, it is possible that error recognition in high-frequency words may be even more difficult than in low-frequency words.

Concerning the frequency effect in visual word recognition, the ERP evidence is relatively consistent. Generally, low-frequency words elicited larger (negative) ERP amplitudes than high-frequency words (Osterhout et al., 1997; Sereno et al., 1998, 2003; Assadollahi and Pulvermüller, 2003; Hauk and Pulvermüller, 2004; Hauk et al., 2006). The frequency effect was found both in the presentation of words in isolation (Sereno et al., 1998) and in the context of sentences (Sereno et al., 2003). Larger amplitudes for low-frequency words reflect the difficulty in accessing their lexical representations (Sereno et al., 2003). Using MEG, Assadollahi and Pulvermüller (2003) showed the dependence of the frequency effect on word length during reading recurrent words: the frequency effect was observed 120–170 ms after stimulus onset for short words of 3–4 letters and after 225–250 ms for long words of 5–7 letters. The localization of the source showed that the effect of frequency was most pronounced over the left occipitotemporal areas (visual word form areas; Assadollahi and Pulvermüller, 2003). Osterhout et al. (1997) reported a frequency effect ranging from 150 to 250 ms during reading normal or scrambled English prose; the average length of their stimuli ranged from 2.5 to 6.2 letters. Hauk et al. (2006) showed an early frequency effect at 110 ms (for words of 3–6 letters) using a visual lexical decision task. In the experiment of Proverbio et al. (2008), the frequency effect was observed later, at around 240 ms, which the authors associated with the presence of long words (8–9 letters) in the stimulus set. Furthermore, low-frequency words showed lower amplitudes than high-frequency ones, which does not align with the results of other studies; the reason for this inconsistency, according to the authors, was the specific nature of the letter detection task (since no semantic analysis was required for the task) they used (Proverbio et al., 2008). Strijkers et al. (2015) showed that the frequency effect was observed in the semantic task as early as 120 ms after stimulus onset. However, the frequency effect emerged at the earliest 200 ms after stimulus onset in the color categorization task with the same words. (Vergara-Martínez et al. (2020a) demonstrated that the word-frequency effect occurred earlier (around 200 ms after stimulus onset) in the go/no-go than in the two-choice response procedure (around 300 ms after stimulus onset). Together, these studies indicate that the frequency effect is found in various paradigms. Although the onset of the frequency effect may depend on the performed task, the frequency effect is a reliable marker for determining lexical access.

Unlike the amplitude effect of word frequency, the timing of the word frequency effect is quite discrepant across the literature, ranging from an early N1-P2 sensitivity (Sereno et al., 1998, 2003; Assadollahi and Pulvermüller, 2003; Dambacher et al., 2006; Hauk et al., 2006; Zhang et al., 2009; Araújo et al., 2015; Eberhard-Moscicka et al., 2016; Wang et al., 2021) to a longest of 300 ms and more (Polich and Donchin, 1988; Hauk and Pulvermüller, 2004; Faísca et al., 2019) even in similar experimental paradigms, indicating that lexical access is located later on in processing. For example, Hauk and Pulvermüller (2004) showed that lower ERP amplitudes in English native speakers during lexical decision tasks were elicited by words with high frequency compared to low-frequency words in the latency ranges 150–190 ms and also in 320–360 ms. The frequency effect has often been associated with lexical-semantic component N400, a negative wave between 300 and 600 ms after the stimulus (Kutas and Federmeier, 2000, 2011), and the N400 amplitude is larger for low-frequency words than high-frequency words (Barber et al., 2004; Kutas and Federmeier, 2011; Vergara-Martínez et al., 2020b).

The pseudohomophone effect was studied using ERP much less often than the frequency effect. This effect is commonly explained by an orthography–phonology conflict (Ziegler et al., 2001; Briesemeister et al., 2009) and is primarily associated with phonological processing. The pseudohomophone effect is found in the N400 window or even later in most studies when smaller negativity is related to higher activation of a particular phonological representation (Kramer and Donchin, 1987; Bentin et al., 1999; Proverbio et al., 2004; Vissers et al., 2006; Briesemeister et al., 2009; González-Garrido et al., 2015; Costello et al., 2021). N400 has a larger amplitude for pseudohomophones than for words (Briesemeister et al., 2009; Hasko et al., 2013; González-Garrido et al., 2015). However, the ERP results show that phonological activation may occur at an early stage of visual word recognition as early as 150 ms (associated with the P2 component) after stimulus onset and may influence lexical access (Braun et al., 2009; Zhang et al., 2009). Unlike the frequency effect, the pseudohomophone effect does not depend on the length of the stimulus (Ziegler et al., 2001; Briesemeister et al., 2009). However, phonological effects can be modulated by the orthographic transparency of the writing system (Simon et al., 2006; Frost and Ziegler, 2007).

We would like to draw attention to the conditions for generating pseudohomophone stimuli, and the frequency of the basic words used to create pseudohomophones in some previous ERP studies. The procedure for creating pseudohomophone stimuli was not always described in detail (e.g., Newman and Connolly, 2004; Grainger et al., 2006; Araújo et al., 2015). In addition, the conditions for creating pseudohomophones could differ even within the same study. To generate pseudohomophones, both one and two letters of the base word were changed (e.g., Braun et al., 2009; Briesemeister et al., 2009; Costello et al., 2021); these were only vowels (e.g., Vissers et al., 2006) or vowels and consonants at the same time (e.g., Briesemeister et al., 2009). Only González-Garrido et al. (2015) mentioned that the stimuli used are the most frequent orthographic errors in Spanish. Taha and Khateb (2013) apparently also used words with real errors in Arabic as stimuli. Some authors pay attention to the fact that their pseudohomophones are not orthographically similar to words (e.g., Newman and Connolly, 2004) to minimize the contribution of orthography to their processing. Using words with fundamental spelling errors as pseudohomophones is likely to complicate recognition, as we often face misspelled words in our daily lives instead of artificially generated pseudohomophones. The frequency of base words to generate pseudohomophones is also often not described (e.g., Vissers et al., 2006; Taha and Khateb, 2013; Kemény et al., 2018). Sauseng et al. (2004); González-Garrido et al. (2015) used base words of medium frequency, and Briesemeister et al. (2009) only low-frequency base words. The frequency of pseudohomophones was taken into account in Braun’s experiment (Braun et al., 2009): the pseudohomophone effect was strongest for stimuli derived from low-frequency base words, a finding consistent with some previous behavioral research (Jared and Seidenberg, 1991). Therefore, we assume that frequency can influence the process of recognizing fundamental spelling errors.

In this study, we used fundamental spelling errors in the Russian language. We constructed all the stimuli by changing only one letter in a similar position in the word; in addition, we used only one type of spelling violation—we were interested in errors in an unstressed vowel. Vowel reduction, that is, a process that neutralizes phonological contrasts between vowels in unstressed syllables, is an essential linguistic phenomenon since vowels are the main syllabic element. Vowel reduction is one of the most characteristic features of stress-timed languages: in English, many vowels in unaccented syllables are reduced to schwa, whereas in Russian, the process appears to be more complex (Jaworski, 2010). It is worth noting that the reduction of unstressed vowels is not displayed in Russian orthography. There are five vowel phonemes in Standard Russian [i, e, a, ɔ, u]. In unstressed syllables, the five-element set is reduced to two subsystems, consisting of three elements each [i, a, u] and [i, ə, u], depending on the position in the word (Kniazev and Pozaritskaya, 2011). The Russian vowels /a/ and /o/ have the same unstressed allophones, and /e/ reduces to [i] in unstressed syllables. The vowel /u/ may also be centralized, but it does not typically merge with any other vowel. We selected words with vowel phonemes [i, e, a, ɔ] in unstressed syllables, which are more susceptible to confusion. For example, the pronunciation of the correct spelled word “вопрос” (“question”) with an unstressed first syllable and the misspelled word “вапрос” (incorrect spelling of a word “вопрос”) is the same [vɐˈpros]. First, this choice was driven by the fact that this is a fundamental type of spelling error for the Russian language: It is pretty complex and widespread. Mistakes in unstressed vowels /i, e, o, a/ are the most common, even among children with a high level of spelling competence and among foreigners studying Russian (Padgett and Tabain, 2005; Oglezneva et al., 2016; Ogneva, 2018). Second, the neural bases for recognizing different types of errors may differ. For example, pseudowords created from high-frequency words by transposing two letters or replacing one letter have different ERP correlates (Vergara-Martínez et al., 2013). Therefore, we have chosen only one type of error, and the misspelled word always differed from the correctly spelled word by only one letter. Third, sound change in which the phonemes /o/ or /e/ are realized as more or less close to [a] and [i] also occurs in other East Slavic languages, e.g., in Slovenian, Bulgarian (Toporišič, 1992; Crosswhite, 2001), implying that these types of spelling error are quite common.

In this study, we used a method of silent reading, in which participants are not required to pronounce words or explicitly decide upon their lexical status. In contrast to the lexical decision task, which is used more often in ERP research on pseudohomophones (e.g., Braun et al., 2009; Briesemeister et al., 2009; González-Garrido et al., 2015; Costello et al., 2021), the silent reading task allows the exploration of cognitive processes underlying reading without extraneous task demands and is better suited for research on visual word recognition (Blythe et al., 2020; Bermúdez-Margaretto et al., 2020a). We manipulated the word form frequency (high vs. low) and the correct spelling (correct words vs. words with error) of the written words in the silent word reading ERP task to investigate whether word frequency influences error recognition. In this study, we aimed to investigate two ERP components, P200 and N400, which are involved in orthographic, phonological, and semantic processing and are often considered together in reading research (Dambacher et al., 2006; Zhang et al., 2009; Bermúdez-Margaretto et al., 2020b; Wang et al., 2021). If error recognition is limited to the orthography stage, we would expect the spelling effect to induce differences only in the early time windows of the ERP. If the process of recognizing errors in words involves phonological and semantic processes, we would also expect the spelling effect to induce differences in late time windows of the ERP. In addition, the frequency effect will indicate at what point lexical access occurs when errors are recognized. Since the ERP task did not allow us to evaluate the speed and accuracy of recognition of correctly spelled words and words with errors, we also performed a second experiment where we used a paradigm similar to the lexical decision task. We expected the effect of frequency on spelling recognition and a faster reaction time and lower error rate for correctly written words than for misspelled words at the behavioral level.

## Materials and Methods

### Participants

Twenty-six native Russian speakers (8 males, 18 females) aged 18 to 39 years old (mean age 24.2, SD 5.3 years) with normal or corrected-to-normal vision participated in the study. All participants were students who had at least 11 years of education or had a university degree (mean 13.7, SD 2.1 years of education). They were all right-handed and did not have any reported neurological disorders or reading and spelling problems. All participants gave written informed consent in accordance with the Declaration of Helsinki; the informed consent form was approved by the Ethics Committee of the Institute of Higher Nervous Activity and Neurophysiology of the Russian Academy of Sciences (IHNA & NPh RAS). All participants completed two tasks in a shielded room: a behavioral task and an ERP task.

### Behavioral Task

#### Stimuli Material

Four types of stimuli were presented: high-frequency words spelled correctly (25 words, HC—high-frequency correctly spelled words), low-frequency words spelled correctly (23 words, LC—low-frequency correctly spelled words), high-frequency words misspelled (25 words, HE—high-frequency words with errors), and low-frequency words misspelled (23 words, LE—low-frequency words with errors). All stimuli were nouns of 5–6 letters long, spelled correctly, and with errors in an unstressed vowel. The mean length of HC words was 5.40 (SD 0.50), LC 5.52 (SD 0.51), HE 5.32 (SD 0.48), LE 5.65 (SD 0.49); the mean number of syllables of HC words was 2.16 (SD 0.37), LC 2.04 (SD 0.21), HE 2.08 (SD 0.28), LE 2.17 (SD 0.39). The error was in the first syllable, except for two words, in which the error was in the second syllable. Example stimuli for each condition are given in Table 1. All stimuli used in this study are shown in the Appendix.

**Table 1:** Examples stimuli for each condition in the behavioral task.

**Word**	***CF***	**Frequency, ipm**	**Length**	**Number of syllables**	**IPA**	**EP**	**Word translation**
*HC words*							
доска		67	5	2	[dɐˈska]		board, plank
спина		183.1	5	2	[spʲɪˈna]		back
борьба		190.5	6	2	[bɐrʲˈba]		struggle, fight
*LC words*							
блоха		4.3	5	2	[bɫɐˈxa]		flea
желток		3.3	6	2	[ʐɨɫˈtok]		yolk, vitellus
вражда		7	6	2	[vrɐˈʐda]		enmity, hostility
*HE words*							
галова	голова	709	6	3	[ɡəɫɐˈva]	2	head
цвиток	цветок	92.4	6	2	[t͡svʲɪˈtok]	3	flower
систра	сестра	121.3	6	2	[sʲɪˈstra]	2	sister
*LE words*							
птинец	птенец	4.6	6	2	[ptʲɪˈnʲet͡s]	3	chick, nestling
бигун	бегун	2.4	5	2	[bʲɪˈɡun]	2	runner
лесица	лисица	2.8	6	3	[lʲɪˈsʲit͡sə]	2	female of the fox

*CF, Correct form for HE and LE words; ipm, instances per million words; IPA, The International Phonetic Alphabet; EP, Error position for HE and LE words*.

All stimuli in this experiment were selected from the Frequency Dictionary of the Modern Russian Language (Lyashevskaya and Sharov, 2009). We took into account the fact that not all low-frequency words are equally difficult; for example, low-frequency compound words can consist of two high-frequency words (Brysbaert et al., 2018). All low-frequency words in behavioral task were not related to high-frequency words through compounding. Since the knowledge of a word by the subject may also be an important factor in addition to the frequency of the word—the so-called variable of word prevalence introduced by Brysbaert et al. (2018)—at the end of the task, we asked the subjects whether they had encountered unfamiliar words. All the words in the behavioral task were familiar to all subjects.

The mean frequency of high-frequency correctly spelled words was 207.77 (range 34.00–926.00) instances per million, 152.70 (range 30.10–709.00) instances per million for high-frequency words with errors, 12.23 (range 0.70–25.80) instances per million for low-frequency correctly spelled words, and 8.61 (range 1.60–23.20) instances per million for low-frequency words with errors. We used the Wilcoxon signed ranks test to confirm that there was no evidence for a reliable difference with respect to word frequency for correctly spelled words and words with errors (*p* > 0.1). HC and HE stimuli differed from LC and LE stimuli in word frequency of occurrence (HC vs. LC, *p* = 0.00003; HE vs. LE, *p* = 0.00003).

#### Experimental Procedure and Analysis

Stimuli were written in white Liberation Sans font 125 pt and were presented in lowercase in the center of the screen on a black background with a viewing distance of approximately 1 m. Stimuli were presented in random order on a 19″ LG FLATRON L1952T monitor using the PsychoPy Experiment Builder v3.0.7 software (Peirce et al., 2019).

In contrast to the ERP task, the subjects had to give an answer regarding the correct spelling of words. Depending on the stimulus, it was necessary to press the left or right buttons of the Logitech F310 gamepad. The button used for each type of response was counterbalanced across the subjects. For 17 subjects, the left button corresponded to the correctly spelled word, and the right button corresponded to the incorrectly spelled word. For 9 subjects, the buttons were interchanged.

A stimulus was presented until the subjects’ response followed by an average interstimulus interval of 1,800 ms (jittered between 1,300 and 2,300 ms). All of the stimuli required a response.

The mean reaction time and error rate for each condition were analyzed using ANOVAs with repeated measures (RM). Since the error rate was very low, the reaction time was evaluated regardless of the correctness of the answer. Given the evidence that effects for stimuli constructed from words of different frequencies in the leading edge may differ from in the bulk of the reaction time distribution (Perea et al., 2005), we also calculated for each subject 0.1 quantile of the reaction time distribution and performed RM ANOVA. The factors were Spelling (correct vs. misspelled) and Frequency (high vs. low). In the behavioral task, the stimuli were simpler (the length was shorter and the frequency was less different between the groups of stimuli) than in the ERP task. We expected behavioral effects to be more pronounced than ERP effects.

### ERP Task

#### Stimuli Material

All stimuli were nouns of 5–7 letters long, spelled correctly, and with errors in an unstressed vowel, only one error was allowed in each misspelled word. Stimuli in the ERP task were similar but not equal to the behavioral task stimuli. There were four types of stimuli: 37 HC words, 38 LC words, 39 HE words, and 39 LE words. The mean length of HC words was 6.32 (SD 0.75), LC 6.26 (SD 0.69), HE 6.31 (SD 0.73), LE 6.35 (SD 0.71); the mean number of syllables of HC words was 2.59 (SD 0.60), LC 2.58 (SD 0.50), HE 2.69 (SD 0.66), LE 2.56 (SD 0.50). The error was in the first or second syllable (11 HE words and 8 LE words). Example stimuli for each condition are given in Table 2.

**Table 2:** Examples stimuli for each condition in the ERP task.

**Word**	***CF***	**Frequency, ipm**	**Length**	**Number of syllables**	**IPA**	**EP**	**Word translation**
*HC words*							
команда		174,2	7	3	[kɐˈmandə]		command, team
момент		306,8	6	2	[mɐˈmʲent]		moment
офицер		118,7	6	3	[ɐfʲɪˈt͡sɛr]		officer
*LC words*							
лосиха		0,6	6	3	[ɫɐˈsʲixə]		a female moose
новатор		2,8	7	3	[nɐˈvatər]		innovator
обивка		2,9	6	3	[ɐˈbʲifkə]		upholstering
*HE words*							
абъект	объект	206,4	6	2	[ɐˈbjekt]	1	object
вапрос	вопрос	805,8	6	2	[vɐˈpros]	2	question
карабль	корабль	112,5	7	2	[kɐˈrablʲ]	2	ship
*LE words*							
абрезок	обрезок	2,7	7	3	[ɐˈbrʲezək]	1	end, shred, snippet
матылёк	мотылёк	2,7	7	3	[mətɨˈlʲɵk]	2	moth, butterfly
гарняк	горняк	2,6	6	2	[ɡɐrˈnʲak]	2	miner

*CF, Correct form for HE and LE words; ipm, instances per million words; IPA, The International Phonetic Alphabet; EP, Error position for HE and LE words*.

All stimuli in this experiment as well as in the behavioral task were selected from the Frequency Dictionary of the Modern Russian Language (Lyashevskaya and Sharov, 2009). All low-frequency words in the ERP task were not related to high-frequency words through compounding. All the words in the ERP task were familiar to all subjects.

Every low-frequency word in the ERP task appears <3 times per million, and every high-frequency word appears >100 times per million. The mean frequency of high-frequency correctly spelled words was 258.62 (range 108.70–926.00) instances per million, 228.38 (range 102.80–805.80) instances per million for high-frequency words with errors, 2.45 (range 0.60–2.90) instances per million for low-frequency correctly spelled words, and 2.70 (range 1.20–2.90) instances per million for low-frequency words with errors. We used the Wilcoxon signed-ranks test to confirm that there was no evidence for a reliable difference with respect to word frequency for correctly spelled words and words with errors (*p* > 0.1). HC and HE stimuli differed from LC and LE stimuli in word frequency of occurrence (HC vs. LC, *p* < 0.0001; HE vs. LE, *p* < 0.0001). We also compared the orthographic neighborhood size using the StimulStat database (Alexeeva et al., 2018); this parameter did not differ statistically for correct and misspelled conditions (*p* > 0.1). The mean orthographic neighborhood size of HC words was 0.70 (SD 1.18), LC 0.55 (SD 1.01), HE 0.54 (SD 1.02), LE 0.33 (SD 0.53). Frequency of bigram contained an error for misspelled words, or bigram contained unstressed vowel, which could have been misspelled for a correctly spelled word was equalized for correct and misspelled words (*p* > 0.1): the mean bigram token frequency of HC words was 2,672,986 (SD 1554942), LC 2496484 (SD 1574369), HE 2375382 (SD 1327010), LE 2491027 (SD 1804774; determined according to Frequency Dictionary of Modern Russian Language, Lyashevskaya and Sharov, 2009).

#### Experimental Procedure

The conditions for presenting stimuli were the same as those described in “Experimental Procedure and Analysis”.

During the EEG recording, the subjects had to silently read the words presented on the screen. A stimulus was shown for 200 ms, followed by an average interstimulus interval of 1,850 ms (jittered between 1,500 and 2,200 ms). All of the stimuli required no response. The 153 stimuli were presented in two blocks, with a short break between blocks.

#### EEG Recording and Analysis

An electroencephalogram was recorded from 19 electrodes Fp1, Fp2, F3, F4, F7, F8, C3, C4, T3, T4, T5, T6, P3, P4, O1, O2, Fz, Cz, and Pz, placed according to the International System 10–20, left and right mastoid electrodes served as reference channels for the monopolar ipsilateral design of EEG recording. Data were sampled at 250 Hz with 0.1–70 Hz filter settings with impedances below 10 kΩ. The offline processing was carried out using Brain Vision Analyzer 2.0.4 (Brain Products, GmbH, Munich, Germany). Eye movements were corrected using an ICA procedure. The data were digitally bandpass filtered (0.3–30 Hz), segmented (−300 to 1,500 ms), artifact rejected (± 100 uV), followed by a visual inspection, and averaged for all of the stimuli in each condition separately. About 5% of the trials were discarded. The averaged data were baseline corrected (300 ms prior to stimulus presentation). ERPs resulted from averaging the segmented trials separately in each condition. There were 34–37 trials included for each condition in the average ERP data from most of the participants.

**Figure 1:**
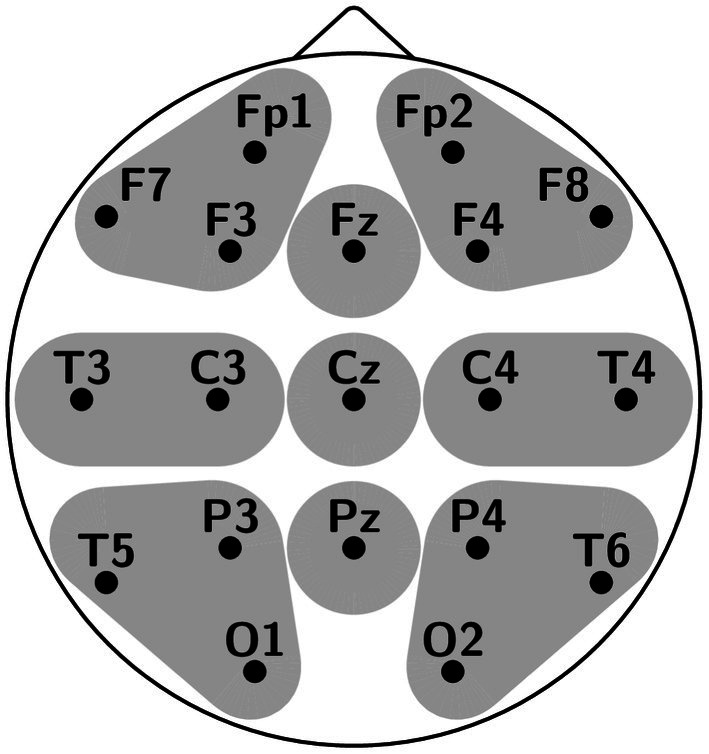
Electrode montage with regions used for analysis.

The analysis was performed in two independent time windows: the 160–280 ms time window (roughly corresponding to P200) and the 350–700 ms time window (roughly corresponding to N400). Basically, we are interested in epoch 350–700 ms. However, it lasts 350 ms, and some subtle effects may be averaged out during analysis. Therefore, we performed RM ANOVAs in two shorter time windows: 350–500 ms and 500–700 ms. RM ANOVAs were applied to the average amplitude of each time window. Scalp electrodes were divided into 9 regions of interest (ROI; Figure 1): left anterior/LA (Fp1, F7, F3), midline anterior/MA (Fz), right anterior/RA (Fp2, F4, F8), left central/LC (T3, C3), midline central/MC (Cz), right central/RC (C4, T4), left posterior/LP (T5, P3, O1), midline posterior/MP (Pz), and right posterior/RP (P4, T6, O2). We averaged the mean ERP amplitude for each ROI over the electrodes in each region. Statistical analysis was performed using the STATISTICA software (Statsoft, Tulsa, OK, United States). The factors were Spelling (correct vs. misspelled), Frequency (high vs. low), Laterality (left vs. midline vs. right), and Anterior–Posterior electrode position (anterior vs. central vs. posterior). We focused on Spelling and Frequency effects and the interactions between these factors. All significant (*p* < 0.05) main and interaction effects were followed by *post-hoc* Bonferroni-corrected contrasts. To correct violations of sphericity and homogeneity, the Greenhouse–Geisser correction was applied as well. Partial eta squared ($\eta_{p}^{2}$) was applied as a measure of effect size, with values of 0.01–0.05 indicating small effects, 0.06–0.13 indicating medium effects, and ≥ 0.14 indicating large effects.

## Results

### Behavioral Data

**Figure 2:**
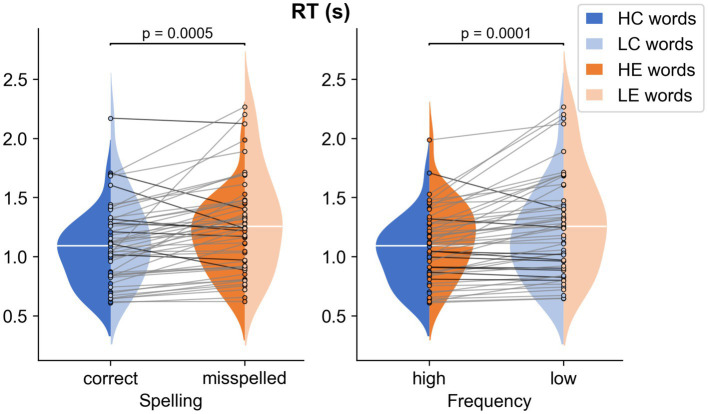
RM ANOVA results for response times (RT). The distributions of the RT for the four types of stimuli are displayed by a violin plot, means are shown as horizontal white lines.

The RM ANOVA results for mean response times are shown in Figure 2. The analysis of mean reaction time indicated a significant main effect both for Spelling *F*(1,25) = 16.01, *р* = 0.0005, $\eta_{p}^{2}$ = 0.39 (correct vs. misspelled, 1.09 vs. 1.26 s) and Frequency *F*(1,25) = 20.48, *р* = 0.0001, $\eta_{p}^{2}$ = 0.45 (high vs. low, 1.09 vs. 1.26 s): The reaction time of low-frequency words was longer than that of high-frequency words, and the reaction time of words with errors was longer than that of correctly spelled words. No significant Spelling × Frequency interaction was found. The analysis of 0.1 quantile of the reaction time distribution showed the same significant main effect both for Spelling *F*(1,25) = 11.19, *р* = 0.003, $\eta_{p}^{2}$ = 0.31 (correct vs. misspelled, 0.74 vs. 0.79 s) and Frequency *F*(1,25) = 14.48, *р* = 0.001, $\eta_{p}^{2}$ = 0.37 (high vs. low, 0.75 vs. 0.78 s).

The error rate of each condition was less than 6%: mean error rate for HC words was 0.77%, for LC words was 1.85%, for HE words was 2.62%, for LE words was 5.29%. Out of 26 participants, 21 achieved 100% accuracy for HC words and 16 achieved 100% accuracy for LC words. No further analysis was performed due to the very low number of errors.

### ERP Data

**Figure 3:**
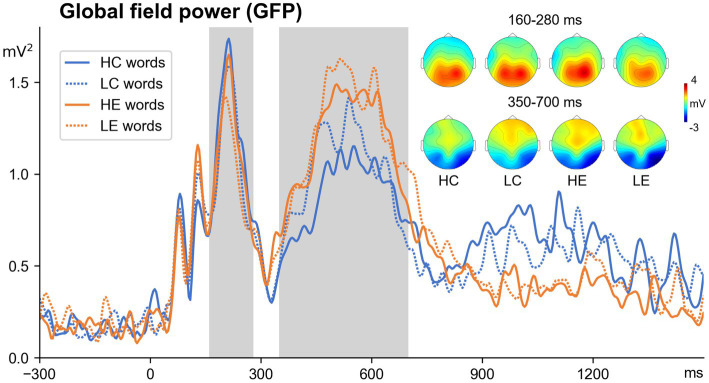
Global field power (GFP, all electrodes) averaged across four experimental conditions and topographic maps.

Two main time windows were selected based on data from previous studies (e.g., Bermúdez-Margaretto et al., 2020a; Wang et al., 2021 for P200; Kutas and Federmeier, 2000, 2011; Szewczyk and Schriefers, 2018, for N400) and of Global Field Power (GFP; see Figure 3), which permits the optimal choice periods of stable topography, i.e., occurrence times of evoked components (Lehmann and Skrandies, 1980). We first computed an average ERP across each condition and participant and subjected this to GFP transformation. We found the main peaks around 160–280 ms and 350–700 ms.

**Figure 4:**
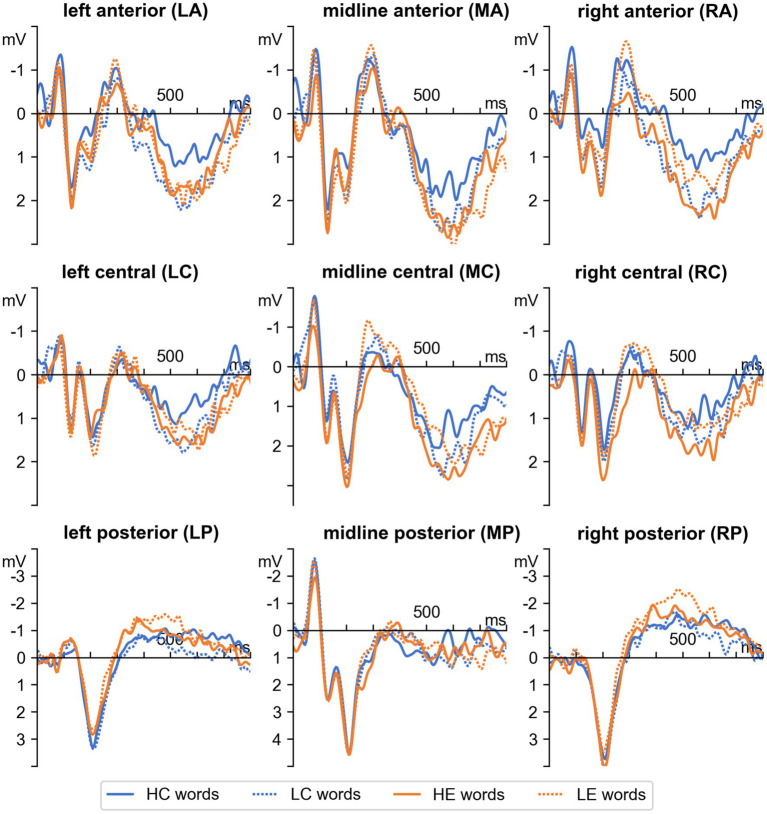
Grand average ERPs for four conditions across all 9 regions of interest.

Figure 4 illustrates the averaged ERPs for four conditions across all 9 regions of interest. As shown in Figure 4, the positive peak was in 160–280 ms (P200) window, and the positive and negative peaks were in the 350–700 ms (P300/N400) time windows. The timing and distribution of these components between all conditions were similar (Figure 3).

In the 160–280-ms time window, a significant Spelling × Anteriority × Laterality interaction [*F*(4,100) = 3.31, *р* = 0.03, $\eta_{p}^{2}$ = 0.12] was found. *Post-hoc* analysis showed that the effect of Spelling was significant in the left posterior region (*p* = 0.0003): The amplitude of P200 was more positive for correctly spelled words than for misspelled words (2.03 vs. 1.59 μV).

In the 350–700-ms time window, a significant Spelling × Frequency interaction [*F*(1,25) = 4.32, *р* = 0.04, $\eta_{p}^{2}$ = 0.15] and a significant Spelling × Frequency × Anteriority × Laterality interaction [*F*(4,100) = 2.54, *р* = 0.04, $\eta_{p}^{2}$ = 0.10] were found. *Post-hoc* analysis revealed that a more positive response was for misspelled words than for correctly spelled words only for high-frequency words in the RA region (*p* < 0.0001, HC vs. HE, 0.71 vs. 1.75 μV) and that more negative response was for misspelled words than for correctly spelled words only for low-frequency words in the LP region (*p* < 0.01, LC vs. LE, −0.31 vs. −1.04 μV), RP region (*p* < 0.001, LC vs. LE, −0.98 vs. −1.83 μV). In addition, low-frequency correctly spelled words elicited a more positive effect than high-frequency correctly spelled words in the LA region (*p* < 0. 01, HC vs. LC, 0.71 vs. 1.54 μV) and RA region (*p* < 0.0001, HC vs. LC, 0.71 vs. 1.65 μV).

In the 350–500-ms time window Spelling × Frequency interaction [*F*(1,25) = 1.82, *р* = 0.18] and Spelling × Frequency × Anteriority × Laterality interaction [*F*(4,100) = 1.94, *р* = 0.11] were not statistically significant. However a significant Spelling × Anteriority interaction [*F*(2,50) = 3.39, *р* = 0.04, $\eta_{p}^{2}$ = 0.12] was found. *Post-hoc* analysis revealed that a more negative response was for misspelled words than for correctly spelled in all posterior regions (LP, MP, RP, *p* < 0.05, 1.52 vs. 2.19 μV).

In the 500–700-ms time window, a significant Spelling × Frequency interaction [*F*(1,25) = 6.43, *р* = 0.02, $\eta_{p}^{2}$ = 0.20] and a significant Spelling × Frequency × Anteriority × Laterality interaction [*F*(4,100) = 2.61, *р* = 0.04, $\eta_{p}^{2}$ = 0.10] were found. *Post-hoc* analysis revealed that a more positive response was for misspelled words than for correctly spelled words only for high-frequency words in the MA region (*p* < 0.001, HC vs. HE, 1.52 vs. 2.33 μV), RA region (*p* < 0.0001, HC vs. HE, 0.92 vs. 2.03 μV), LC region (*p* < 0.01, HC vs. HE, 0.63 vs. 1.35 μV), MC region (*p* < 0.0001, HC vs. HE, 1.50 vs. 2.46 μV), RC region (*p* < 0.01, HC vs. HE, 0.74 vs. 1.50 μV) and that more negative response was for misspelled words than for correctly spelled words only for low-frequency words in the LP region (*p* < 0.05, LC vs. LE, −0.15 vs. −0.84 μV), RP region (*p* < 0.001, LC vs. LE, −0.80 vs. −1.63 μV). In addition, low-frequency correctly spelled words elicited a more positive effect than high-frequency correctly spelled words in the LA region (*p* < 0. 01, HC vs. LC, 0.90 vs. 1.65 μV), MA (*p* < 0.05, 1.52 vs. 2.19 μV) region and RA region (*p* < 0.0001, HC vs. LC, 0.92 vs. 1.82 μV). Thus, in the 500–700-ms time window, we got almost the same effects as in the large time window of 350–700 ms.

## Discussion

We investigated the time course of recognition of the most frequent orthographic errors in Russian (error in an unstressed vowel in the root) and the effect of word frequency on this process using the behavioral and the ERP tasks. In the behavioral task, we did not find the effect of frequency on the error recognition process. However, ERP data demonstrate that the neuronal underpinnings of recognizing errors in words during reading may depend on word frequency.

Behavioral data showed both the frequency effect (faster RT for high-frequency words than for low-frequency words) and the spelling effect (faster RT for correctly spelled words than for misspelled words). Both of these findings are consistent with the well-known word recognition effects (e.g., Monsell et al., 1989; Bentin and Ibrahim, 1996; Rayner, 1998; Proverbio et al., 2004; Yan et al., 2006; Faísca et al., 2019; Laurinavichyute et al., 2019). The slow processing of misspelled words compared to correctly spelled words can have several possible explanations. Rahmanian and Kuperman (2019) suggested that words with spelling errors may form their orthographic representations in the mental lexicon since they occur in naturally produced written language. However, correctly spelled words have orthographic representations that we encounter more often and that are more familiar to us, and thus we recognize them faster. Nevertheless, the possible interference of phonology cannot be ruled out. Words with errors in an unstressed vowel and correctly spelled words sound the same, and the orthographic decision task probably is not pure of an orthographic measure because not only orthographic but also phonological activation is higher for words than for pseudohomophones (Rastle and Brysbaert, 2006; Montant et al., 2011). According to dual-route theory for pseudohomophones there is a conflict between the two routes because the phonological route provides evidence in favor of word representations, whereas no orthographic representation is found (Braun et al., 2009). Thus, slow misspelled word processing also can result from this conflict since the phonological representations of such words do not coincide with their spelling representations in memory as opposed to correctly spelled words. Although we did not statistically analyze the error rate, it was highest for low-frequency misspelled words, which indicates the complexity of these stimuli for subjects. It is probably related to more blurry representations in memory for lower frequency words than for higher frequency words in which the subjects were almost not mistaken. However, it is essential to note that we found no effect of frequency on error recognition for either the mean reaction time or the leading edge of the reaction time distribution. On the one hand, this may indicate that error recognition speed does not depend on word frequency. On the other hand, a possible explanation for this might be that in the behavioral task, the stimuli were simpler than in the ERP task. In addition, behavioral measures are less sensitive than ERP and may not reveal some effects.

The present ERP data demonstrate a spelling effect already ~200 ms after stimulus presentation. We found that correctly spelled words induce a larger P200 in the left posterior region than misspelled words. In the literature, P200 has been associated with word-form encoding processes and the extraction of the orthographic and phonological features of a word in the early stages of word processing (Barnea and Breznitz, 1998; Carreiras et al., 2005; Dambacher et al., 2006; Wang et al., 2021). Modulation of the P200 component was found for known versus novel written words, and an increase in the amplitude of the P200 component when learning novel written words has been associated with a modification of the sublexical orthographic process and switching from letter-by-letter decoding to a more holistic lexical-type access of newly formed representations (Bermúdez-Margaretto et al., 2020b). In a study of Arabic spelling errors, a larger P200 amplitude was in words than in pseudohomophones, which the authors explained by the difference in orthographic analysis when a word is recognized as a familiar orthographic pattern (Taha and Khateb, 2013). It was also shown that P200 could be modulated by orthography alone during reading (Kong et al., 2012), and it reflects automatic sublexical processing indexing initial discrimination of word stimuli (Comesaña et al., 2012). We suppose the large P200 amplitude for correctly spelled words compared to misspelled words likely reflects greater sensitivity to familiar spelling patterns. The absence of frequency effect in this time window indicates that these are lower-level spelling processes such as whole word orthography processing that do not involve lexical access. Note that misspelled words in unstressed vowels sound like actual words; therefore, we cannot completely exclude the influence of phonology. Nevertheless, previous studies of the time course of orthography and phonology demonstrated that orthographic codes are activated very early, and phonological activation begins after that (Grainger et al., 2006; Carreiras et al., 2009); therefore, we expected the influence of phonology at the later stages.

To better understand what processes occur in the later time window of 350–700 ms, we divided it into two parts: the traditional N400 epoch (350–500 ms) and the 500–700 ms epoch, which is associated with a slow positive wave. The scalp distribution of these components in all three time periods was similar: we observed a positive wave in the frontocentral regions, along with a negative wave in the parietal–temporal-occipital areas (see Figures 3–4). It has been demonstrated in the past literature that the N400 component found in lexical or semantic categorization tasks is overlapped by the modulation of P300, a component associated with attention mechanisms activated to perform a task (e.g., Polich, 1985, 2004; van Hees et al., 2017; Alday and Kretzschmar, 2019; Bermúdez-Margaretto et al., 2019). Indeed, the ERP signal at any point in time may be composed of multiple components; however, the approach based only on waveform component structure may lead to inconsistent results (Brouwer and Crocker, 2017). Therefore, in addition to the analysis in the time window of 350–700 ms, we performed the analysis in two shorter time windows without considering the waveform. The current study found that almost the same frequency effects were observed in the short 500–700-ms time window and in the large time window of 350–700 ms: high-frequency misspelled words elicited a more positive wave than high-frequency correctly spelled words, and low-frequency misspelled words elicited a more negative wave than low-frequency correctly spelled words. In addition, in the 350–500-ms time window, we found a more negative response for misspelled words than correctly spelled words in parietal–temporal-occipital regions regardless of word frequency, i.e., the same pattern observed in a later time window for only low-frequency words. Therefore, we assumed this negative pattern might result from a broadly distributed N400 effect that overlaps with a frontocentral P300. In the following paragraphs, we discuss the results in the time window of 350–700 ms in more detail.

We found a more positive response for misspelled words than for correctly spelled words only for high-frequency words in the right anterior region. This spelling effect for high-frequency words was most pronounced between 500 and 700 ms. The P300 wave is not a component exclusively related to language processing; however, it is found in any psycholinguistic paradigm that requires an assessment of stimulus and a binary decision (Proverbio et al., 2009; González-Garrido et al., 2014; Alday and Kretzschmar, 2019). This wave reflects an information-processing cascade when attentional and memory mechanisms are engaged, namely, final stimulus evaluation (Polich, 1985, 2004; Taylor, 1993). This component can also reflect lexical decisions based on orthographic properties (Mariol et al., 2008; Kriukova and Mani, 2016). Although our passive reading task did not require making any decisions, it may involve involuntary attention processes and categorization of correctly spelled words and words with errors. Our behavioral data demonstrated that words with errors are difficult for subjects, so these stimuli impose greater demands on attention resources, which result in larger P300 amplitude. This finding is consistent with studies on stimuli with orthographic violations (e.g., Newman and Connolly, 2004; Mariol et al., 2008; González-Garrido et al., 2014) in which the P300 amplitude was higher for words or pseudowords with violations; however, these studies did not take into account word frequency. Taylor (1993) also noted the relation of this wave with orthography: the P300 wave was observed in the orthographic and phonological tasks, but the P300 amplitude was lower in the phonological task. Moreover, differences in P3a amplitude, a subcomponent of the P300, are observable with familiar lexical stimuli when they are orthographically unexpected (Savill and Thierry, 2011), such as misspelled words in our experiment. We assume that the P300 effect may reflect mainly orthographic processing of high-frequency misspelled and correctly spelled words and categorization processes based on orthography. Importantly, simultaneously with the P300 effect, the frequency effect was observed. That is, this stage involves accessing a lexical-semantic representation. According to the dual-route model of reading, both direct and indirect routes can serve for semantic access (Coltheart et al., 1993; Coltheart, 2000). Through the direct route, sublexical orthographic information makes direct contact with whole-word orthographic representations. As a result, access to whole-word phonology on the one hand and higher-level semantic information on the other is provided (Grainger and Ziegler, 2011). It is generally agreed that pseudohomophone stimuli involve some form of sublexical conversion of print-to-sound; that is, they activate the indirect path (Grainger and Ziegler, 2011). Both direct and indirect routes include the sublexical and the lexical level, and at each level, there is an orthographic and a phonological layer. At the lexical level, these layers may interact through bidirectional connections (Diependaele et al., 2010). In general, this means the processing of misspelled high-frequency and low-frequency words should involve common phonological processes. The observed N400 differences for correct spelled and misspelled words in the time window of 350–500 ms could be attributed to activations of phonological representations for misspelled words regardless of the frequency of base words (more about the N400 effect below). However, for high-frequency words, shortly after this, a transition to the orthographic layer occurs when the phonological processing of those high-frequency words is inhibited. The P300 differences, which are observed only for high-frequency words, might be due to the transition from phonological to orthographic processes.

In contrast to high-frequency words, there was no P300 effect for low-frequency stimuli. However, we found that a more negative response in the left posterior and right posterior regions corresponding to the N400 wave was for misspelled words than for correctly spelled words and the N400 effect was more prolonged for low-frequency words. Previous studies have reported the association of N400 with phonological processing (Barnea and Breznitz, 1998; Zhang et al., 2009; Wang et al., 2021). Barnea and Breznitz (1998) found longer latencies and higher amplitudes of N400 during the phonological task (rhyme judgment) compared to the orthographic task (orthographic similarity/dissimilarity judgment). Interestingly, no N400 effect was detected when studying Arabic spelling errors, although the late positive wave differences between correct spelled words and misspelled words were revealed, and the authors interpreted this result as evidence of careful orthographic analysis without involving phonological processing (Taha and Khateb, 2013). However, in most studies using pseudohomophones, phonological activation in visual word recognition and pseudohomophone effect has been associated with the N400 component, but not P300 (Kramer and Donchin, 1987; Bentin et al., 1999; Proverbio et al., 2004; Vissers et al., 2006; Briesemeister et al., 2009; González-Garrido et al., 2015; Costello et al., 2021). N400 has a larger amplitude for pseudohomophones than for words (Briesemeister et al., 2009; Hasko et al., 2013; González-Garrido et al., 2015). Misspelled words are similar to pseudohomophone stimuli; they are visually similar to correctly spelled words and are phonologically identical. Phonological plausibility causes a conflict that impedes spelling recognition, and its resolution requires repeated access to the memory, where the visual representation of the word is stored. However, for misspelled words, orthographic representation in memory may be blurry or absent. For words presented in isolation, the N400 wave is associated with lexical–semantic processing, and the modulation of its amplitude reflects processing costs during the retrieval of properties related to a word form stored in memory (Kutas and Federmeier, 2011). The conflict caused by phonological similarity is probably more pronounced for low-frequency words and is associated with the longer modulation of the N400 component for misspelled words. The greater conflict for low-frequency words can be explained in the context of dual-route theory (Coltheart et al., 2001). For low-frequency words, orthographic activation will be weaker than for high-frequency words, which means that activation of the phonological route will increase. One can expect that low-frequency misspelled words will provide stronger phonological activation and cause greater conflict than high-frequency misspelled words.

Importantly, phonological processing is considered slower than orthographic processing (Barnea and Breznitz, 1998). However, we observed both the N400 and P300 effects for words of different frequencies in the same time window, which seems to reflect the temporal overlap between phonological processes for low-frequency words and categorization processes based on orthographic properties for high-frequency words. Furthermore, this assumption may be indirectly confirmed by the fact that the reaction times for high-frequency misspelled words and low-frequency misspelled words in the behavioral task did not differ. N400 can also reflect categorization processes, and some authors emphasize that, given the possible confusion of effects, it is necessary to draw careful conclusions about the processes under study (Bermúdez-Margaretto et al., 2019). On the other hand, evidence suggests that the P300 and N400 components are primarily independent and reflect two separate but interacting processes (Alday and Kretzschmar, 2019). Harm and Seidenberg (2004) proposed a cooperative division of labor between phonological and orthographic pathways to meaning activation, and word frequency may alter the relative contribution of the two routes. Our data may indicate that at the late stage of error recognition in words of different frequencies, the contribution of these pathways may differ. It could mean that recognition of high-frequency misspelled words and high-frequency correctly spelled words shifts from phonological to orthographic processes, while low-frequency misspelled words are accompanied by more prolonged phonological activation, which can be reflected in different ERP waves.

Another important finding of this study is that the frequency effect has been identified in the 350–700-ms time window and was especially strong in the 500–700-ms time window: low-frequency correctly spelled words elicited a more positive response than high-frequency correctly spelled words in anterior regions. Generally, lower amplitudes for words with higher frequencies have been reported, and these results are well described (Osterhout et al., 1997; Sereno et al., 1998, 2003; Assadollahi and Pulvermüller, 2003; Hauk and Pulvermüller, 2004; Hauk et al., 2006). As for the timing of the word frequency effect, we expected it earlier than 350–700 ms because we used stimuli similar in length to words used in studies in which the frequency effect was observed in the time range of the P200 component (150–250 ms, e.g., Osterhout et al., 1997; Assadollahi and Pulvermüller, 2003; Dambacher et al., 2006). The frequency effect is a marker of lexical access; higher frequency words elicit a smaller amplitude than words of lower frequency, suggesting that semantic access is easier for more frequently encountered words (Van Petten and Kutas, 1990; Barber et al., 2004; Vergara-Martínez and Swaab, 2012). However, specific features of the task can affect the occurrence time of the frequency effect (Strijkers et al., 2015). Perhaps, spelling errors in words complicate lexical access; therefore, there is no difference between high-frequency and low-frequency words in the early stages.

In conclusion, our ERP results indicate that the spelling effect already occurs ~200 ms after stimulus presentation regardless of the frequency of base words, and at the later stage, this effect is modulated by the frequency of the base words. Considering our results in the context of a dual-route model, we concluded that recognizing misspelled high-frequency and low-frequency words involves common orthographic and phonological processes associated with P200 and N400 components such as whole word orthography processing and activation of phonological representations correspondingly. However, at the 500–700 ms stage (associated with lexical-semantic access), error recognition depends on the word frequency. One possible explanation for these differences could be that at the 500–700 ms stage recognition of high-frequency misspelled and correctly spelled words shifts from phonological to orthographic processes, while low-frequency misspelled words are accompanied by more prolonged phonological activation. We believe these processes may be associated with different ERP components P300 and N400, reflecting a temporal overlap between categorization processes based on orthographic properties for high-frequency words and phonological processes for low-frequency words. One of the limitations of the current study is that we used very few electrodes, precluding any possibility of source localization of ERP generators. Another potential limitation of our study is that the stimuli in the behavioral task and the ERP task slightly differed from each other, which may explain some of the inconsistency in the results of these tasks. In addition, unlike the ERP task in the behavioral task, we did not control such parameters as orthographic neighborhood size and bigram frequency for correct and misspelled conditions.

## Data Availability Statement

The raw data supporting the conclusions of this article will be made available by the authors, without undue reservation.

## Ethics Statement

The studies involving human participants were reviewed and approved by The Ethics Committee of the Institute of Higher Nervous Activity and Neurophysiology of the Russian Academy of Sciences. The patients/participants provided their written informed consent to participate in this study.

## Author Contributions

EL and OM developed the general idea for the study. EL collected the data, performed the analysis, and wrote the paper. OM oversaw all stages of data analysis and edited the article. All authors contributed to the article and approved the submitted version.

## Funding

This study was partially supported by Grant No. 20-013-00514 of the Russian Foundation of Basic Research (RFBR) and funds within the state assignment of the Ministry of Education and Science of the Russian Federation for IHNA & NPh RAS.

## Conflict of Interest

The authors declare that the research was conducted in the absence of any commercial or financial relationships that could be construed as a potential conflict of interest.

## Publisher’s Note

All claims expressed in this article are solely those of the authors and do not necessarily represent those of their affiliated organizations, or those of the publisher, the editors and the reviewers. Any product that may be evaluated in this article, or claim that may be made by its manufacturer, is not guaranteed or endorsed by the publisher.
